# The normal ranges of cardiovascular parameters measured using the ultrasonic cardiac output monitor

**DOI:** 10.14814/phy2.13195

**Published:** 2017-03-21

**Authors:** Giles N. Cattermole, P. Y. Mia Leung, Grace Y. L. Ho, Peach W. S. Lau, Cangel P. Y. Chan, Stewart S. W. Chan, Brendan E. Smith, Colin A. Graham, Timothy H. Rainer

**Affiliations:** ^1^ Emergency DepartmentCentre Hospitalier Universitaire de KigaliKigaliRwanda; ^2^ Department of MedicineAustin/Northern HealthMelbourneAustralia; ^3^ Department of MedicineWestern HealthMelbourneAustralia; ^4^ Accident and Emergency DepartmentQueen Elizabeth HospitalHong Kong SAR; ^5^ Accident and Emergency Medicine Academic UnitPrince of Wales HospitalThe Chinese University of Hong KongHong Kong SAR; ^6^ School of MedicineUniversity of Notre Dame AustraliaSydneyNew South WalesAustralia; ^7^ Intensive Care UnitBathurst Base HospitalBathurstNew South WalesAustralia; ^8^ University Hospital of WalesHeath ParkHeathCardiffU.K.

**Keywords:** Diagnostic techniques and procedures, Doppler, hemodynamics, normal ranges, ultrasonography

## Abstract

The ultrasonic cardiac output monitor (USCOM) is a noninvasive transcutaneous continuous wave Doppler method for assessing hemodynamics. There are no published reference ranges for normal values in adults (aged 18–60 years) for this device. This study aimed to (1) measure cardiovascular indices using USCOM in healthy adults aged 18–60 years; (2) combine these data with those for healthy children (aged 0–12), adolescents (aged 12–18), and the elderly (aged over 60) from our previously published studies in order to present normal ranges for all ages, and (3) establish normal ranges of USCOM‐derived variables according to both weight and age. This was a population‐based cross‐sectional observational study of healthy Chinese subjects aged 0.5–89 years in Hong Kong. USCOM scans were performed on all subjects, to produce measurements including stroke volume, cardiac output, and systemic vascular resistance. Data from previously published studies (children, adolescents, and the elderly) were included. Normal ranges were defined as lying between the 2.5th and 97.5th percentiles. A total of 2218 subjects were studied (mean age = 16.4, range = 0.5–89; 52% male). From previous studies, 1197 children (aged 0–12, 55% male), 590 adolescents (aged 12–18, 49% male), and 77 elderly (aged 60–89, 55% male) were included. New data were collected from 354 adults aged 18–60 (47% male). Normal ranges are presented according to age and weight. We present comprehensive normal ranges for hemodynamic parameters obtained with USCOM in healthy subjects of all ages from infancy to the elderly.

## Introduction

The ultrasonic cardiac output monitor (USCOM1A; USCOM Pty Ltd., Coffs Harbour, NSW, Australia) provides a rapid noninvasive measure of hemodynamic parameters using continuous wave Doppler ultrasound (CW Doppler) (USCOM Ltd., 2006a,b).

There is increasing interest in measuring flow‐based hemodynamic parameters such as cardiac output, (CO), cardiac index (CI), stroke volume (SV), systemic vascular resistance (SVR), oxygen delivery (DO_2_), and oxygen consumption (VO_2_) as these are considered to be not only basic physiological parameters in health, but also indicators of illness, where they have been shown to be more effective predictors of response to therapy than static measures such as blood pressure or central venous pressure (CVP) (Marik et al. 2011).

The emergence of noninvasive devices has opened the door for measuring hemodynamics in many research and clinical settings. Noninvasive techniques are at least as accurate as traditional invasive thermodilution methods, as well as being more practical and safer. Since its introduction in 2001, the USCOM has been used in a wide range of clinical settings, including critical care, anesthesiology, emergency medicine, obstetrics, and neonatology. Its accuracy and reliability have been validated in animal and human studies against alternative methods including pulmonary artery thermodilution (Jain et al. 2008; Stewart et al. 2008; Dhanani et al. 2011; Phillips et al. 2012; McNamara et al. 2014). There is widespread support for the use of Doppler‐based ultrasound methods to assess hemodynamics for fluid management in resuscitation and perioperatively (Brierley et al. 2009; Thiel et al. 2009; Kuper et al. 2011; NHS Technology Adoption Centre 2013).

For any method of clinical measurement, it is important to establish the normal ranges of values obtained using that method in order to determine the significance of any subsequent individual measurement.

Normal ranges for hemodynamic variables obtained with USCOM have been published for neonates, children, adolescents, and the elderly (Cattermole et al. 2010; He et al. 2011; Ho et al. 2013; Chan et al. 2014). However, there are no published ranges for adults, nor have ranges been derived according to subject weight, and no unifying overview of changes in hemodynamics from the first year of life to the elderly. The objectives of this study were therefore:

1. To measure hemodynamic indices using USCOM in healthy adult ethnic Chinese aged 18–60 years.
2. To combine these data with those for healthy children (aged 0–12), adolescents (aged 12–18), and the elderly (aged over 60) from our previously published studies (Cattermole et al. 2010; Ho et al. 2013; Chan et al. 2014), in order to present normal ranges for all ages.
3. To establish and compare normal ranges of USCOM‐derived hemodynamic variables according to weight and age.

## Methods

### Ethical approval

The Clinical Research Ethics Committee of the Chinese University of Hong Kong approved the study (reference number CRE‐2009.482). Signed informed consent was obtained from all adult subjects.

This cross‐sectional observational study of 18‐ to 60‐year‐olds was conducted in Hong Kong between September 2010 and May 2011 concurrent with the adolescent study (Ho et al. 2013). Data were obtained from other studies performed between October 2008 and January 2009 (children) (Cattermole et al. 2010), and between February and October 2012 (elderly) (Chan et al. 2014).

### Participants and setting

Healthy Chinese adults aged 18–60 were recruited. Children and adolescents were recruited from local schools as described previously (Cattermole et al. 2010; Ho et al. 2013). Adults were recruited through St. John's Ambulance personnel. The elderly were recruited through friends and colleagues of staff (Chan et al. 2014). Exclusion criteria included lack of consent, acute or chronic illness, smoking, and current use of any medication. Non‐Chinese subjects were excluded. Similarly, only healthy Chinese subjects from previously published studies were included in the analysis.

### Sample size of current study

Estimated means and standard deviations for SV were used to calculate a sample size of 105 subjects in each subgroup to achieve 95% confidence that the true mean lies within 5% of that observed. To define up to three age or weight subgroups (according to the centile curves produced), 315 subjects were required.

### Measurements

USCOM is a direct derivative of echocardiography which uses CW Doppler ultrasound to provide measurements of hemodynamic indices. Using either the suprasternal insonation window for the aortic valve, or the left parasternal window for the pulmonary valve, the device measures the velocity time integral (VTI) of the ejection flow and heart rate (HR). A proprietary algorithm based on height (in subjects greater than 50 cm) or weight (less than 50 cm) is used to derive the cross‐sectional area (CSA) of the two valves, and stroke volume is calculated as SV = CSA × VTI. Heart rate is calculated from the interval between systolic ejections, while concurrent systolic and diastolic blood pressure values (SBP, DBP) are entered manually, from which mean arterial pressure (MAP) is calculated as MAP = DBP + ([SBP − DBP]/3). From these data, USCOM derives values for cardiac output (CO = SV × HR), systemic vascular resistance (SVR = MAP/CO), and other hemodynamic parameters as shown in Table 1. Flow time (FT) is the systolic ejection time in milliseconds. Stroke volume variation is defined as (SVmax − SVmin × 100)/([SVmax + SVmin]/2). Currently, the USCOM measures or derives 22 hemodynamic variables simultaneously. Body surface area (BSA) is calculated by the USCOM using the formula of Du Bois and Du Bois (1916), from which BSA‐indexed values for CO, SV, and SVR were derived (CI, SVI, and SVRI).

**Table 1:** USCOM‐derived hemodynamic parameters

Parameter	Unit	Definition/equation
Preload		
Flow time (FT)	Ms	Systolic ejection time
Flow time corrected (FTc)	Ms	${\text{FT}}_{c}=\text{FT}/\sqrt{\left(R-\text{R interval}\right)}$
Stroke volume variation (SVV)	%	$\text{SVV}=\left({\text{SV}}_{\max}-{\text{SV}}_{\min}\times 100\right)/\left[\left({\text{SV}}_{\max}+{\text{SV}}_{\min}\right)/2\right]$
Contractility		
Heart rate (HR)	min^−1^	Number of cardiac cycles in beats per min
Velocity time integral (VTI)	m	VTI=∫FT/0V(t)dt
Stroke volume (SV)	ml	$\text{SV}=\text{VTI}\times \pi r^{2}$
Stroke volume index (SVI)	ml·m^−2^	SVI=SV/BSA
Afterload		
Systemic vascular resistance (SVR)	d·s·cm^−5^	SVR=(MAP−CVP)×80/CO
Systemic vascular resistance index (SVRI)	d·s·cm^−5^·m^2^	SVRI=SVR×BSA
Tissue perfusion		
Cardiac output (CO)	L·min^−1^	CO=SV×HR
Cardiac index (CI)	L·min^−1^.m^−2^	CI=CO/BSA

BSA, body surface area (m^2^); CVP, central venous pressure (mmHg); MAP, mean arterial pressure (mmHg); R–R interval, heart beat periodicity (s); *π*r^2^, cross‐sectional area of outflow tract (m^2^).

Operators were trained to use the USCOM, and data collection followed the same procedure as described previously (Cattermole et al. 2010; Ho et al. 2013; Chan et al. 2014). Standing height was measured barefoot to the nearest 0.1 cm using a measuring tape (range = 0.0–200.0 cm). Body weight was measured to the nearest 0.2 kg using electronic calibrated scales (Compact Precision Scale C200H, Conair Far East Ltd., Hong Kong, China). Blood pressure was then measured with an appropriately sized cuff using automated oscillometry (Patient Monitor BX‐10ne, Omron Healthcare Co. Ltd., Kyoto, Japan), with the subject supine and at rest immediately prior to USCOM examination.

In this study, following a short period of rest and with the subject lying supine, the suprasternal insonation window was used to obtain measurements of aortic valve flow. A minimum of three consecutive Doppler ejection profiles of diagnostic quality were required for each measurement, and three measurements were made for each subject. For analysis, the single best quality trace was used (as defined previously according to the signal characteristics of the flow tracing; Cattermole et al. 2009).

### Statistical analysis

LMS Chartmaker Pro v2.3 software (Cole and Pan, Medical Research Council UK, 2006) was used to describe the data in centile curves (2.5, 10, 50, 90, 97.5). The relationships between USCOM‐derived hemodynamic indices and both age and weight were modeled by the LMS method of Cole and Green (1992). Briefly, the relationship is described by three age‐specific cubic spline curves known as L, M, and S. M represents the median, S is the coefficient of variation, and L is the Box–Cox transformation that renders the data to follow a normal distribution, conditional on age. Combination of these three functions generates centile values for each parameter. Age and weight subgroups were defined according to the curves produced.

MedCalc v14.12.0 (MedCalc Software bvba, Belgium) was used for descriptive analysis of the data within weight and age subgroups. The Shapiro–Wilk test was used to determine the normality of the distribution. Data were analyzed using medians and ranges, or means and standard deviation, as appropriate. Comparison of groups was made with the appropriate parametric or nonparametric tests.

## Results

There was a total of 2218 subjects (mean age = 16.4, range = 0.5–89; 52% male). From the previous studies, 1197 children (aged 0–12, 55% male), 590 adolescents (aged 12–18, 49% male), and 77 elderly (aged 60–89, 55% male) were included. New, and previously unpublished, data were collected from 354 adults (aged 18–60, 47% male).

Under 18 years, none of the data were normally distributed, while in adults the majority of variables were abnormally distributed.

Under 18 years, there were no significant gender differences for any variables. Over 18 years there were small but statistically significant differences between male and female. Because these differences were largely attributable to weight, and for ease of use, the rest of the data are presented without reference to gender.

**Figure 1:**
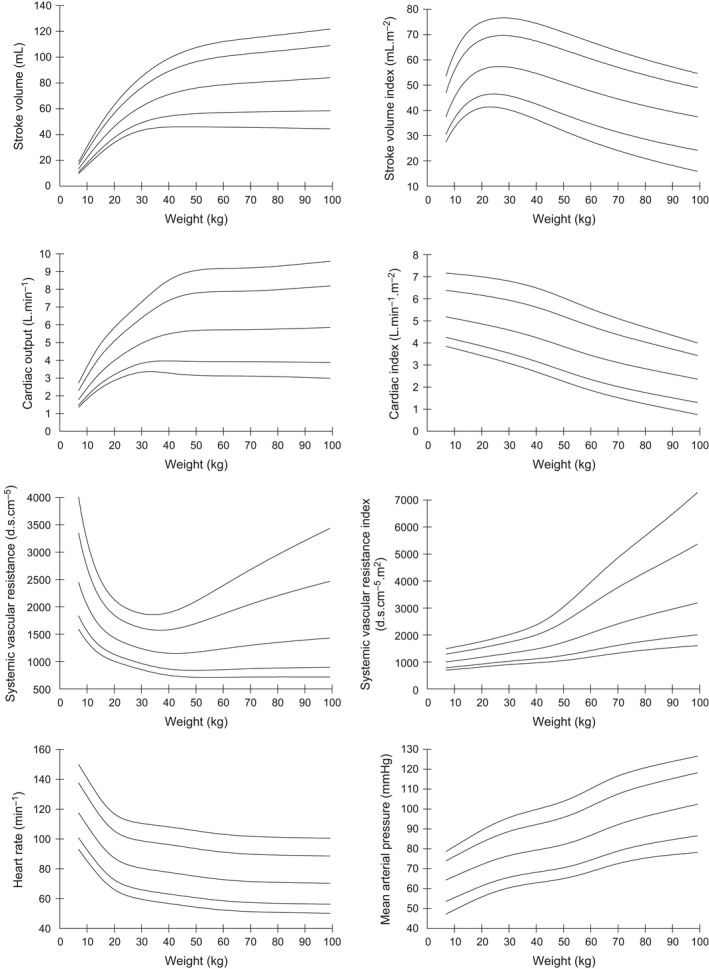
Centile curves (2.5, 10, 50, 90, 97.5) of hemodynamic parameters with weight.

**Figure 2:**
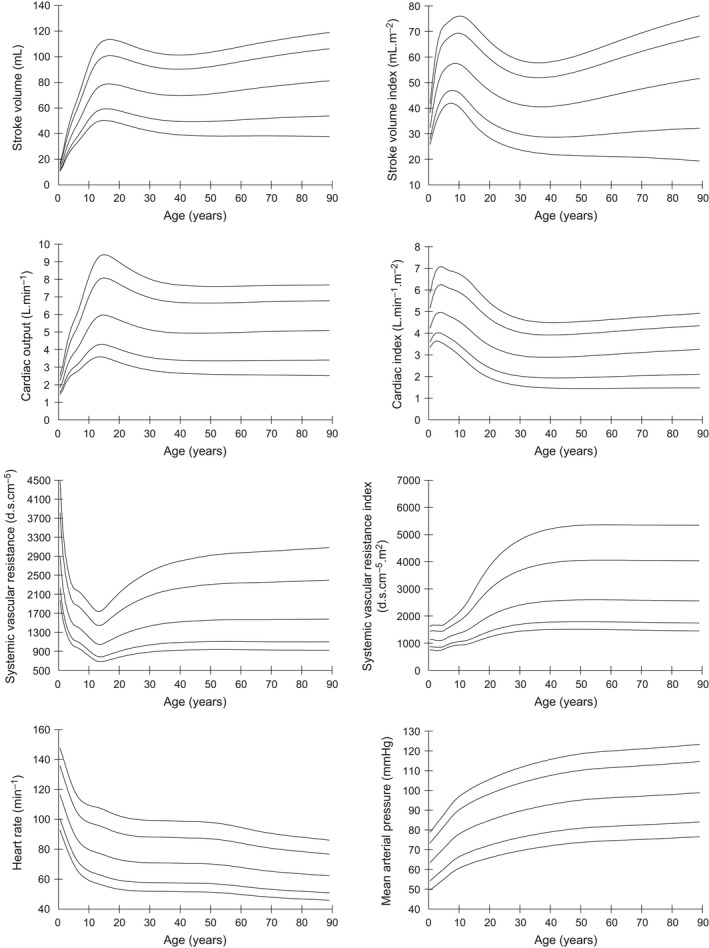
Centile curves (2.5, 10, 50, 90, 97.5) of hemodynamic parameters with age.

Centile curves (2.5, 10, 50, 90, 97.5) for stroke volume (SV), stroke volume index (SVI), cardiac output (CO), cardiac index (CI), systemic vascular resistance (SVR) and systemic vascular resistance index (SVRI), mean arterial pressure (MAP), and heart rate (HR) against each of age and weight are presented in Figures 1 and 2. Based on these curves, two adult age groups were defined: 18–30 and 30–60 years. Weight subgroups were similarly defined, using data from all studies.

Medians and 2.5%–97.5% ranges were used to present normal ranges in age and weight subgroups, for each of the USCOM variables as shown in Tables 2 and 3.

**Table 2:** Normal ranges for hemodynamic parameters according to age

	0–2.9 years	3–5.9 years	6–11.9 years	12–17.9 years	18–29.9 years	30–59.9 years	60 + years
*n*	65	353	773	460	267	223	77
MAP (mmHg)	66.7 (50.7–80.0)	69.3 (52.7–87.2)	77.3 (60.6–96.0)	80.7 (65.3–103.7)	84.3 (66.1–105.6)	94.0 (71.4–118.3)	96.0 (71.7–120.0)
HR (min^−1^)	113.9 (79.4–146.3)	92.9 (71.8–123.1)	80.8 (59.8–111.4)	78.6 (56.6–106.5)	71.6 (53.3–100.9)	71.3 (53.0–93.7)	64.4 (46.4–89.7)
FT (ms)	283.3 (220.3–362.1)	316.7 (250.0–366.7)‐	326.7 (280.0–376.7)	311.4 (244.5–370.0)	312.5 (241.0–372.7)	355.7 (260.3–449.3)	395.0 (303.6–476.8)
FTc (ms)	386.2 (296.4–449.5)	388.0 (325.2–466.1)	379.2 (318.7–440.5)	357.0 (274.7–423.1)	338.2 (248.8–415.0)	385.0 (297.4–476.9)	409.3 (341.5–490.1)
SVV (%)	9.7 (1.0–77.4)	10.6 (1.6–29.0)	8.7 (1.4–23.4)	16.8 (4.5–36.3)	19.1 (7.2–44.8)	21.8 (7.2–56.4)	14.8 (5.3–55.4)
VTI (m)	23.8 (17.6–34.0)	28.6 (22.0–38.2)	30.2 (22.5–39.8)	29.5 (18.7–40.0)	26.8 (17.4–37.1)	25.3 (13.7–34.6)	31.0 (16.3–42.2)
SV (mL)	23.0 (14.0–43.0)	40.1 (26.0–58.0)	59.2 (39.1–94.2)	79.0 (46.0–114.9)	76.3 (48.2–114.3)	68.8 (39.1–98.5)	81.8 (39.7–115.3)
SVI (mL.m^−2^)	44.0 (30.4–63.9)	53.3 (40.0–71.0)	56.6 (42.3–76.0)	52.8 (31.1–72.7)	47.1 (26.9–68.2)	40.3 (23.2–56.6)	51.1 (27.9–72.0)
SVR (d.s.cm^−5^)	2045 (1188–3627)	1496 (1003–2359)	1282 (809–1899)	1043 (686–1883)	1244 (795–2226)	1566 (906–2950)	1435 (1015–3027)
SVRI (d.s.cm^−5^.m^2^)	1103 (698–1647)	1123 (740–1668)	1350 (913–2082)	1567 (963–3327)	2070 (1286–3838)	2686 (1499–5073)	2446 (1504–4940)
CO (L.min^−1^)	2.63 (1.61–3.90)	3.68 (2.52–5.34)	4.82 (3.10–7.84)	6.23 (3.61–9.73)	5.42 (3.06–9.00)	4.88 (2.51–7.77)	5.22 (2.97–7.49)
CI (L.min^−1^.m^−2^)	4.71 (3.41–6.42)	4.97 (3.55–6.90)	4.57 (3.04–6.68)	4.13 (2.17–6.42)	3.33 (1.74–5.48)	2.82 (1.49–4.69)	3.30 (1.88–4.71)

Ranges presented as median (2.5%–97.5% range). Parameters as listed in Table 1.

**Table 3:** Normal ranges for hemodynamic parameters according to weight

	<10 kg	10–14.9 kg	15–19.9 kg	20–29.9 kg	30–49.9 kg	50–74.9 kg	75 + kg
*n*	18	96	299	451	577	686	91
MAP (mmHg)	63.8 (na)	69.5 (51.3–83.8)	69.3 (53.3–89.3)	74.0 (56.3–92.3)	79.3 (65.3–100.3)	86.7 (67.3–113.3)	94.7 (75.2–119.6)
HR (min^−1^)	121.0 (na)	102.4 (79.1–128.5)	90.7 (70.3–119.4)	82.7 (62.0–112.1)	78.7 (56.1–111.9)	72.2 (52.9–100.9)	71.2 (51.1–100.9)
FT (ms)	256.7 (na)	296.7 (229.3–354.3)	316.7 (250.0–370.0)	326.7 (273.3–366.7)	326.7 (268.9–396.9)	322.9 (246.1–438.4)	321.4 (243.1–405.3)
FTc (ms)	365.4 (na)	385.7 (314.7–458.7)	387.5 (326.8–468.3)	382.0 (317.6–446.0)	373.6 (311.8–440.1)	358.5 (269.2–458.7)	349.9 (256.9–451.0)
SVV (%)	9.0 (na)	10.6 (1.0–84.5)	10.4 (1.5–27.6)	9.0 (1.3–24.6)	12.4 (2.5–36.0)	18.2 (5.3–44.9)	18.8 (8.9–50.0)
VTI (m)	20.6 (na)	26.0 (19.1–34.5)	28.5 (21.9–37.1)	29.6 (22.6–38.9)	30.2 (19.8–40.0)	27.9 (16.2–40.2)	26.0 (13.3–39.3)
SV (mL)	16.3 (na)	30.5 (19.2–45.1)	40.9 (28.8–56.5)	53.1 (37.8–76.9)	71.4 (45.9–98.0)	77.7 (44.2–114.7)	77.8 (42.2–115.6)
SVI (mL.m^−2^)	37.0 (na)	49.3 (33.7–68.8)	54.0 (40.3–74.6)	56.5 (42.1–76.9)	55.4 (35.3–72.9)	47.1 (26.3–68.8)	39.3 (21.1–58.7)
SVR (d.s.cm^−5^)	2271 (na)	1767 (1101–2693)	1538 (1073–2228)	1340 (895–1918)	1145 (750–1879)	1263 (725–2517)	1337 (676–2898)
SVRI (d.s.cm^−5^.m^2^)	1056 (na)	1072 (693–1563)	1142 (775–1654)	1250 (833–1754)	1451 (998–2609)	2071 (1166–4407)	2703 (1328–5604)
CO (L.min^−1^)	2.04 (na)	2.97 (2.20–4.85)	3.63 (2.63–5.22)	4.42 (3.04–6.72)	5.56 (3.34–8.36)	5.51 (3.00–9.35)	5.71 (2.69–9.79)
CI (L.min^−1^.m^−2^)	4.43 (na)	5.01 (3.70–7.55)	4.93 (3.60–6.72)	4.69 (3.35–6.87)	4.37 (2.43–6.29)	3.37 (1.83–5.68)	2.91 (1.39–4.91)

Ranges presented as median (2.5–97.5% range). Parameters as listed in Table 1.

## Discussion

This is the first study of normal ranges for USCOM in adults, and the first presentation of ranges for use in all ages from infancy through the elderly. Consistent with our previous studies, all subjects were healthy Hong Kong Chinese. Previously published USCOM values for children and adolescents did not include velocity time integral, flow time, and stroke volume variability, which are presented here for all ages. In addition, collation of the data into a single set permits the generation of smoother and more precise centile curves using the LMS method.

This is also the largest study of hemodynamic values obtained in healthy subjects by any method. This knowledge of healthy normal ranges should be helpful both in physiological research and clinical practice.

Our results are consistent with other published ranges (De Simone et al. 1997). It is impractical and inappropriate to perform large‐scale studies of healthy subjects using pulmonary artery catheterization, precluding direct comparison of derived ranges. However, data for stroke volume index measured using the Fick method were published in 1955 for 67 healthy adult males, which are very similar to ours with mean SVI = 48.9 mL.m^−2^ for 20–30 years and 44.2 mL.m^−2^ for 30–60 years (Brandfonbrener et al. 1955).

This study was not performed to validate the accuracy or reliability of USCOM. Other workers have shown that the USCOM is at least as good as other hemodynamic measurement methods, both invasive and noninvasive (Jain et al. 2008; Stewart et al. 2008; Dhanani et al. 2011; Phillips et al. 2012; McNamara et al. 2014). Our previous studies have demonstrated good interobserver reliability (Cattermole et al. 2010; Ho et al. 2013), but this was in children and adolescents. Others have shown that in the ethnic Chinese population, interobserver reliability decreases with increasing age, associated with increased difficulty of insonation and poor quality scans (Huang and Critchley 2013). These difficulties arise from anatomical changes in the suprasternal notch with increasing age, which impair proper transducer alignment with the aortic valve. This is an important point to note when using USCOM in older subjects; however, in this study, poor quality scans were excluded from the analysis.

For any technology, notwithstanding discussion as to absolute accuracy, it is essential to know the normal ranges obtained with that method. It is likely that different methods will produce consistent over‐ or underestimation of values in comparison with each other, a systematic bias. This does not preclude their scientific and clinical utility, provided that the normal ranges are known for that specific technology.

Normal ranges are conventionally presented as the range within which 95% of values lie. If the data are normally distributed, this is presented as mean ± 1.96 standard deviation. In this study, most data were not normally distributed, so medians with 2.5 and 97.5 centiles are presented.

We have presented normal ranges according to both age and weight. Our preference would be to use weight‐dependent ranges, partly because this is likely to be more important than age physiologically, but also pragmatically as the centile curves are smoother and easier to use. The extent to which age and weight independently contribute to the changes in hemodynamic variables is unclear. Similarly, gender is another potential factor, but the differences observed in age‐dependent values were clinically small even if statistically significant. The gender differences were minimal after accounting for weight. In keeping with previously published ranges, we have not added the further complication of separate ranges for gender.

As might be expected, the data show that heart rate falls and blood pressure rises with age, more rapidly in childhood. This is partly accounted for by the rapid change in height in the first 20 years, with later arterial stiffening accounting for the ongoing rise in blood pressure, reflected by the increasing vascular resistance index seen throughout life.

Cardiac output rises steadily to reach a peak in the teenage years, after which it gradually declines. However, when considered as cardiac output per square meter of body surface area, CI, then cardiac output peaks in 3–6 years old children equivalent to a body weight of 10–15 kg. It is likely that this represents the maximum oxygen requirements of the tissues during periods of maximum growth. The higher absolute cardiac output in the teenage years probably represents a period where growth and activity combine to produce maximum oxygen consumption. From late teens onwards there is little or no growth, but a steadily declining level of physical activity.

Overall, stroke volume rises steadily until late teens then tends to decline gradually over the years, but when viewed against weight, it shows a more linear relationship. Stroke volume index follows a broadly similar pattern to cardiac index peaking between 6 and 12 years of age, and at a body weight of around 30 kg. Unfortunately, our data did not include lean body mass calculation, so it is unknown whether this would be a better reference parameter than either weight or age. As the metabolic requirements of fat are significantly lower than those of the vital organs and muscle, it would seem that lean body mass should be a more accurate reference. Whether this proves to be any better than the traditional standard of body surface area‐indexed measurements would require further studies.

### Limitations

There were very few children in the youngest age group and under 10 kg. Although the centile curves are less reliable therefore, we felt it important to include these groups separately as it was clear their values were significantly different from children in the next age and weight groups.

Our study did not include any subjects over 100 kg nor over 89 years old. From the centile curves it is unlikely that extrapolation beyond 89 years will result in significant inaccuracy. Although in Western societies there are subjects with body weights in excess of several hundred kilograms, it is unlikely that extrapolation to such extremes is necessary given the very low perfusional requirements of body fat. However, it could be useful to study hemodynamics–weight relationships in heavier subjects, given the increasing incidence of obesity in the world.

Our study included only Hong Kong Chinese, but previous comparison with Australian data has not shown clinically significant differences between our populations when adjusted for morphometry (Cattermole et al. 2010; Ho et al. 2013).

This study utilized only the suprasternal approach, measuring flow through the aortic valve. We did not use the pulmonary valve approach in order to maintain consistency with the pediatric and adolescent studies where only aortic valve measurements were used. It is known that it can be more difficult to obtain good quality traces via the suprasternal approach in older Chinese subjects which might increase discomfort (Huang and Critchley 2013). The elderly study had included both aortic and pulmonary valve approaches, but found only small differences between the two: stroke volume was approximately 4% lower using the aortic approach, while heart rate was only slightly higher. It is unlikely therefore that patient discomfort caused by using the suprasternal approach leads to any significant adrenergic‐induced measurement error. In addition, we have reported that in children there is little discomfort from suprasternal USCOM relative to measuring blood pressure oscillometrically (Chan et al. 2013). To minimize anxiety, subjects rested before the scan and were fully informed of what was involved. However, we cannot exclude a small but real effect on hemodynamics resulting from discomfort, but this is likely to be far less than the hemodynamic changes induced by other measurement methods such as pulmonary artery catheters or arterial pulse wave integration.

A further potential problem with the aortic valve approach is the possibility of aortic stenosis. (The same is equally true of the pulmonary valve and pulmonary stenosis.) This is easily identified as one of the measures reported by USCOM is the mean pressure gradient (Pmn) across the valve being examined. The presence of valvular stenosis is therefore not only identified qualitatively, but the severity of stenosis can be quantified from the value of Pmn. In our study, no subject had evidence of aortic stenosis, but for practical use, even if aortic stenosis is present then it does not preclude hemodynamic measurements via the pulmonary valve, as combined stenosis of the aortic and pulmonary valves is extremely rare.

## Conclusions

We have presented comprehensive means and normal ranges for hemodynamic parameters obtained with USCOM in healthy subjects of all ages from infancy to the elderly. We have demonstrated how these parameters vary with age and weight. These ranges represent a reference standard for further physiological research, as well as being of potential value in clinical practice.

## Conflict of Interest

G. N. C. has received support for travel and USCOM presentation from Pacific Medical (the distributors of USCOM in Hong Kong), none since 2012. C. A. G. and T. H. R. have received educational support to develop hemodynamics courses, and the long‐term loan of USCOM machines for clinical and research use. No authors have received any fees either from USCOM Ltd. or distributors. The remaining authors have no conflicts of interest.
